# Identification of microRNAs in Silver Carp (*Hypophthalmichthys molitrix*) Response to Hypoxia Stress

**DOI:** 10.3390/ani11102917

**Published:** 2021-10-09

**Authors:** Qiaoxin Wang, Xiaohui Li, Hang Sha, Xiangzhong Luo, Guiwei Zou, Hongwei Liang

**Affiliations:** 1 College of Fisheries and Life Science, Shanghai Ocean University, Shanghai 201306, China; wangqx1997@163.com; 2 Yangtze River Fisheries Research Institute, Chinese Academy of Fishery Sciences, Wuhan 430223, China; lixiaohui@yfi.ac.cn (X.L.); sh1812@yfi.ac.cn (H.S.); lxz@yfi.ac.cn (X.L.)

**Keywords:** silver carp, *Hypophthalmichthys molitrix*, high-throughput sequencing, microRNA, hypoxia stress

## Abstract

**Simple Summary:**

Hypoxia stress is one of the main problems in silver carp (*Hypophthalmichthys molitrix*) culture. Severe hypoxia stress can lead to damage and even death of silver carp. Therefore, it is very important to explore how silver carp adapt to and respond to hypoxia stress. MicroRNAs play an important role in a series of important life activities in organisms. In this study, the differentiallyexpressed miRNAs were screened from a mixed pool of liver, brain, heart and gill of silver carp under different levels of hypoxia stress by high-throughput sequencing. Our findings provided new insights to further study the miRNA regulatory mechanism and molecular characteristics of anoxic response in silver carp.

**Abstract:**

Hypoxia is one of the serious stresses in fish culture, which can lead to physical and morphological changes, and cause injury and even death to fish. Silver carp (*Hypophthalmichthys molitrix)* is an important economic fish and widely distributed in China. MicroRNA is a kind of endogenous non-coding single-stranded small RNA, which is involved in cell development, and immune response and gene expression regulation. In this study, silver carp were kept in the closed containers for hypoxia treatment by spontaneous oxygen consumption. The samples of heart, brain, liver and gill were collected, and the total RNAs extracted separately from the four tissues were mixed in equal amounts according to the concentration. Afterwards, the RNA pool was constructed for high-throughput sequencing, and based on the small RNA sequencing, the differentially expressed microRNAs were identified. Furthermore, their target gene prediction and enrichment analyses were carried out. The results showed that a total of 229 known miRNAs and 391 putative novel miRNAs were identified, which provided valuable resources for further study on the regulatory mechanism of miRNAs in silver carp under hypoxia stress. The authors verified 16 differentially expressed miRNAs by qRT-PCR, and the results were consistent with small RNA sequencing (sRNA-seq). The predicted target genes number of differentially expressed miRNAs was 25,146. GO and KEGG functional enrichment analysis showed that these target genes were mainly involved in the adaption of hypoxia stress in silver carp through biological regulation, catalytic activity and apoptosis. This study provides references for further study of interaction between miRNAs and target genes, and the basic data for the response mechanism under hypoxia stress in silver carp.

## 1. Introduction

The phenomenon of dissolved oxygen (DO) under 2 mg/L in the water environment is called hypoxia [1]. Hypoxia is an increasing threat factor to aquaculture and can lead to physical and morphological changes, and cause injury and even death to fish [2,3]. In the process of aquaculture, due to high temperature and high cultivation density, the dissolved oxygen in the water is often lower than the normal level, posing a serious threat to the cultured animals [4,5]. Like other fish, silver carp are prone to suffer from hypoxia stress [6]. Hypoxia can cause apoptosis of hepatocytes and compromise the central nervous system and immune system of fish, leading to death [7]. So far, there have been many studies on the effects of hypoxia on fish species, such as the function of zebrafish (*Danio rerio*) miRNA in cellular adaptation under hypoxia [8].

Silver carp, *Hypophthalmichthys molitrix*, is widely distributed in various water bodies in China and is a very popular food because of its fresh meat and rich nutrition. In past years, silver carp rapidly became one of dominant species in aquaculture due to its superior characteristics such as a short food chain, strong disease resistance and low price [9]. Furthermore, it also has a positive effect on improving water quality and plays a vital role in water purification fisheries because it feeds on plankton in water and controls the blue-green algae bloom [10]. However, the hypoxia tolerance of silver carp is very poor, and hypoxia stress usually leads to physical damage and even death due to high-density culture and transportation [11,12]. Hypoxia has become one of the main reasons restricting the survival rate of cultured silver carp. Therefore, studying the hypoxia resistance of silver carp is not only of great significance to the healthy development of silver carp aquaculture, but also has a deeper understanding of the molecular mechanism of hypoxia tolerance of all fish.

The microribonucleic acids (miRNAs) are a kind of endogenous non-coding single-stranded small RNAs with approximately 18–23 nucleotides in length, which are bound to the 3′untranslated region (UTR) of the target gene, thereby repressing and degrading the translation of the target gene mRNA, and playing an important regulatory role [13,14,15]. MiRNAs exist widely in animals, and participate in a series of important life activities of organisms, such as growth and development, apoptosis and immunity [16]. In the largemouth bass (*Micropterus salmoides*), the miRNA–mRNA was comprehensively analyzed by high-throughput sequencing technology and 13 differential expressions miRNAs of liver involved in glucose and lipid metabolism were identified under acute hypoxia [17]. The miR-462/miR-731 cluster of zebrafish was significantly up-regulated under hypoxia stress, and cell-cycle progression was blocked to inhibit cell proliferation and induce apoptosis [8]. In nile tilapia (*Oreochromis niloticus*), miR-204, as an endogenous regulator of vascular endothelial growth factor (VEGF) expression, was inhibited under hypoxia stress, and the VEGF expression level was significantly up-regulated [18]. Recently, a total of 324 miRNAs, including 309 conserved miRNAs and 15 novel miRNAs, were identified by deep sequencing of tested tissues (kidney, spleen, muscle and liver) from the blunt snout bream (*Megalobrama amblycephala*) [19]. High-throughput sequencing technology of miRNAs is an effective method to investigate the expression of miRNAs [20]. Some recent studies on aquatic animals showed that miRNAs were also involved in the process of adapting to changes in the external environment [21]. MiRNA could respond to low salinity stress in the oyster (*Crassostrea gigas*) [22], heat stress of the sea cucumber (*Apostichopus japonicus*) [20], and the adaptation of Pacific whiteleg shrimp (*Litopenaeus Vannamei*) to hypoxia stress [21]. However, the roles of miRNAs under hypoxia stress have not been discussed yet in silver carp.

In this study, we employed a high-throughput sequencing technique to determine the expression of miRNAs in silver carp under hypoxia stress. Gene ontology and KEGG pathway analyses of putative target genes were also carried out. In addition, we also analyzed differentially expressed miRNAs. The data obtained help to clarify the regulatory role of miRNAs in the hypoxic stress response of silver carp, and provide new insights for the study of miRNAs regulation and molecular adaptation mechanisms of silver carp under hypoxia stress.

## 2. Materials and Methods

### 2.1. Ethics Approval

All experimental procedures were conducted according to guidelines of the appropriate Animal Experimental Ethical Inspection of Laboratory Animal Centre, Yangtze River Fisheries Research Institute, Chinese Academy of Fishery Sciences (approval number 2020098).

### 2.2. Experimental Fish and Sample Collection

Silver carp (53.64 ± 4.84 g) in this study were taken from Yaowan Experimental Farm, Yangtze River Fishery Research Institute, Chinese Academy of Fisheries Sciences. The silver carp were temporarily cultured in a tank for a week. Before the experiment, the experimental fish were transferred to the 52.5 L tank for 24 h, and the normal aeration was carried out to maintain the oxygen concentration in the water. A total of 120 healthy silver carp were selected and divided into a normoxia group (T0) and three hypoxia stress groups (T1, T2, T3). They were placed in white water tank (50 cm × 35 cm × 30 cm), and 10 fish were placed in each tank, with 3 replicates in each group. Normal aeration was maintained in the normoxic group (all the fish breathed normally, recorded as T0, dissolved oxygen 6.45 mg/L); hypoxia stress experimental group stopped aeration, sealed water tank with plastic film, respectively. Silver carp gasping for air period (most fish tried to breathe directly through their mouths, recorded as T1, 4 h after the beginning of the experiment, dissolved oxygen 0.76 mg/L), semi-asphyxia period (half of fish lost balance, recorded as T2, 5 h after the beginning of the experiment, dissolved oxygen 0.58 mg/L), asphyxiation period (half of fish sank without the rhythmical opening and closing of the gill flaps, recorded as T3, 6 h after the beginning of the experiment, dissolved oxygen 0.21 mg/L). The fish were sampled, and three silver carp were collected in each water tank at each experiment point after MS-222 (100 mg/L) anesthesia. The heart, liver, brain and gill were kept in 2 mL cryopreservation tube and put into liquid nitrogen, and then stored at −80 °C.

### 2.3. Total RNA Extraction, Library Construction and Small RNA Sequencing

Total RNA was extracted from the heart, liver, brain and gill by Trizol Reagent (Invitrogen). RNA degradation and contamination was monitored on 1% agarose gels. RNA purity was checked using the NanoPhotometer^®^ spectrophotometer (IMPLEN, München, Germany). RNA concentration was measured using Qubit^®^ RNA Assay Kit in Qubit^®^ 2.0 Flurometer (Life Technologies, CA, USA). RNA integrity was assessed using the RNA Nano 6000 Assay Kit of the Bioanalyzer 2100 system (Agilent Technologies, Santa Clara, CA, USA). Finally, the total RNA extracted from four tissues of different groups was mixed in equal amounts to construct the RNA pools of the normoxic group and the hypoxic stress groups.

The sequencing libraries were further constructed using NEBNext^®^ Multiplex Small RNA Library Prep Set for Illumina^®^ (NEB, San Diego, CA, USA) following manufacturer’s instructions. Briefly, RNA adapters were ligated to 3′ and 5′ end of RNA followed by cDNA synthesis and PCR amplification. The cDNA library was separated by PAGE gel, and small RNAs of 18–40 bp were isolated and purified. The clustering of the index-coded samples was performed on a cBot Cluster Generation System using TruSeq PE Cluster Kit v3-cBot-HS (Illumia) according to the manufacturer’s instructions (Figure S3). After the clusters were generated, the libraries were sequenced on the Illumina Noveseq platform to generate 50 bp single-end reading codes.

### 2.4. Identification and Analysis of Differentially Expressed miRNAs

After sequencing, clean reads were obtained by removing reads containing adapter, ploy-N and low quality reads (reads having >50%, bases with quality score ≤5) from raw data. Only the remaining reads were regarded as clean reads and used in following analysis. Finally, clean reads were used for subsequent data analysis. Clean reads were compared to the miRNA sequences specified in the miRbase(v22) database by bowtie2 (2.2.2) software to identify known miRNAs. Using DESeq2 to assess differentially expressed miRNAs, miRNAs with |log2(fold change)| > 1 and adjusted *p*-value < 0.05 were considered significant (Figure S1). Target genes of miRNAs were predicted by MiRanda (v3.3a) and qTar software. The final target set was the intersection of the two tools. Subsequently, GO and KEGG enrichment analysis was performed on the target genes of miRNAs to determine the main biochemical metabolic pathways and signal transduction pathways involved. Only GO terms with a Bonferroni-corrected *p* value ≤ 0.05 and pathways with a FDR ≤ 0.05 were regarded as significantly enriched.

### 2.5. Real-Time PCR Validation

RNA samples from the normal oxygen group and hypoxia stress group were subjected to stem-loop qPCR analysis. MiRNA 1st Strand cDNA Synthesis Kit (Vazyme, Nanjing, China) was used to remove genomic DNA from total RNA and reverse transcribe into cDNA. Quant Studio 5 system was used for amplification reaction, and the amplification system is as follows: 2×ChamQ Universal SYBR qPCR Master Mix (Vazyme, Nanjing, China) 10 μL, forward primer (10 μmol/L) 0.4 μL, reverse primer (10 μmol/L) 0.4 μL, template cDNA 1 μL, ddH_2_O 8.2 μL. The qRT-PCR conditions were carried out as follows: initial denaturation at 95 °C for 30 s, followed by 40 cycles of 30 s denaturation at 95 °C, annealing at 60 °C for 30 s and extension at 72 °C for 20 s. The specificity of PCR amplification was verified by melting curve. U6 was used as an internal control and Premier 5.0 software was used for primer design; stem-loop primer sequence and qPCR primer sequence are listed in Table 1. In addition, 16 differentially expressed miRNAs were randomly selected and their relative expression levels were determined by qRT-PCR to verify the reliability of Illumina Noveseq high-throughput sequencing results. The relative expression levels of miRNAs were calculated by 2^−ΔΔCt^ method [14]. SPSS software, version 26.0 (IBM Corp, Armonk, NY, USA) was used for the statistical analysis.

## 3. Results

### 3.1. Analysis of miRNA Library Sequencing Data

Four miRNA libraries of silver carp (T0, T1, T2, T3) were sequenced with the Illumina Noveseq platform. By high-throughput sequencing, 26,475,225, 26,143,905, 22,941,194 and 28,984,723 raw reads were generated in T0, T1, T2 and T3 group, respectively. After data filtering, 25,558,042, 25,542,062, 19,466,537 and 28,030,238 clean reads with high quality were obtained, accounting for 96.54 %, 97.70 %, 84.85 % and 96.71 % of total reads, respectively (Figure S2). The data quality of the small RNA library was listed in Table 2. 

**Figure 1:**
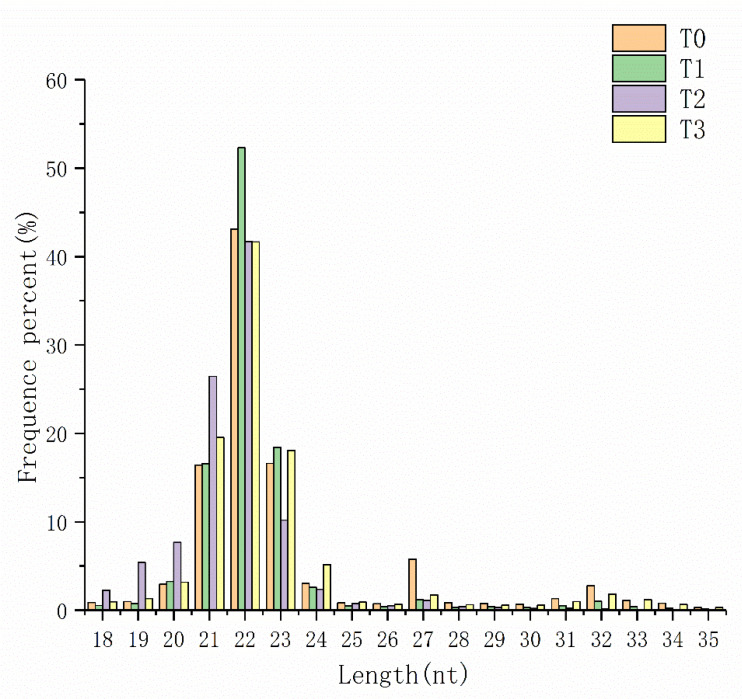
Distribution statistics of small RNA fragment length in four samples. X-axis represents the length distribution of small RNA in T0, T1, T2 and T3 group. The Y axis represents the percentage of each length of small RNA. MiRNAs of 22 nt in length were the most common type.

The clean reads of each sample were screened for small RNA in a certain length range, and it was found that the sequence length was mainly concentrated between 21–23 nt, of which 22 nt accounted for the largest proportion (Figure 1). A total of 288, 286, 249 and 280 known miRNAs were identified in T0, T1, T2, and T3 group, respectively, while 305, 279, 165 and 298 novel miRNAs were predicted respectively (Table S1). The expression levels of these miRNAs are different, but several miRNAs, such as dre-miR-100-5p, dre-let-7e, dre-miR-101a, dre-miR-143 and dre-miR-146a, were all highly expressed in different levels of hypoxia stress (Table 3). According to the exon, intron and repeat sequence information in genome annotation, and the sRNA information in mirBase and Rfam databases, the small RNA was classified and annotated (Table 4). 

### 3.2. Differential Expression Analysis of miRNAs

The transcripts detected with at least 2-fold differences are classified as differential expression miRNAs analysis (|log2Fold Change| ≥ 1, *p* ≤ 0.05). By pairwise comparison of T0 and T1, T0 and T2, T0 and T3, T1 and T2, T1 and T3, T2 and T3, the number of up-regulated differential miRNAs (log2(fold change) > 1 and adjusted *p*-value < 0.05) was 75, 210, 104, 183, 100 and 144, respectively. The number of down-regulated differential miRNAs (log2(fold change) < −1 and adjusted *p*-value < 0.05) was 55, 112, 60, 152, 95 and 186, respectively (Table 5). In addition, some novel miRNAs were also found to be differentially expressed in pairwise comparisons among each group (novel-94, novel-93, novel-899, novel-889, novel-753, novel-683, novel-577).

In addition, the up-regulated miRNAs, such as dre-miR-9-5p, dre-miR-129-5p and dre-miR-499-5p, and the down-regulated miRNAs, such as dre-miR-183-5p, dre-miR-9-4-3p, all showed very high expression levels (|log2Fold Change| > 10).

### 3.3. Validation of Selected miRNAs by Real-Time PCR

**Figure 2:**
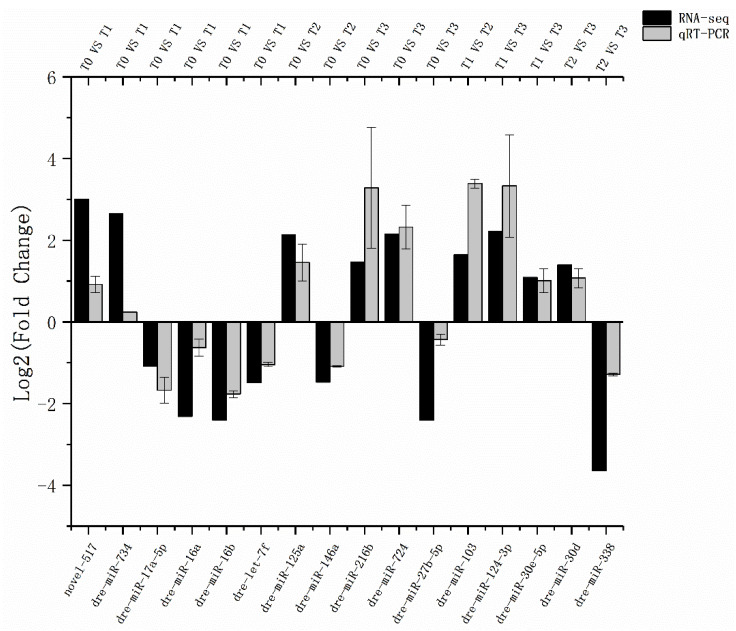
Differently expressed miRNAs validated by qRT-PCR. Comparison was carried out between sRNA-Seq results and qRT-PCR validation results. The 16 selected miRNAs showed concordant expression patterns when the 2 different methods were used.

In order to validate the results of high-throughput sequencing, 16 miRNAs were randomly selected from different groups for real-time PCR. The results showed that the expression trends of these miRNAs were basically consistent with the high-throughput sequencing results, indicating that the sequencing results were highly reliable and could be used for subsequent analysis (Figure 2).

### 3.4. Target Gene Prediction and Functional Annotation of Differentially Expressed miRNAs

MiRanda and qTar software were used to predict the target genes of differentially expressed miRNAs in silver carp under hypoxia stress, and the corresponding number of miRNA target genes was 25,146. The number of target genes for known miRNAs ranged from 29 (dre-miR-736) to 996 (dre-miR-17a-3p), while the number of target genes for novel miRNAs ranged from 39 (novel-878) to 1140 (novel-602). Among these target genes, a series of genes related to hypoxia regulation, such as hypoxia-inducible factor-1alpha, vascular endothelial growth factor and hypoxia up-regulated protein, are included.

**Figure 3:**
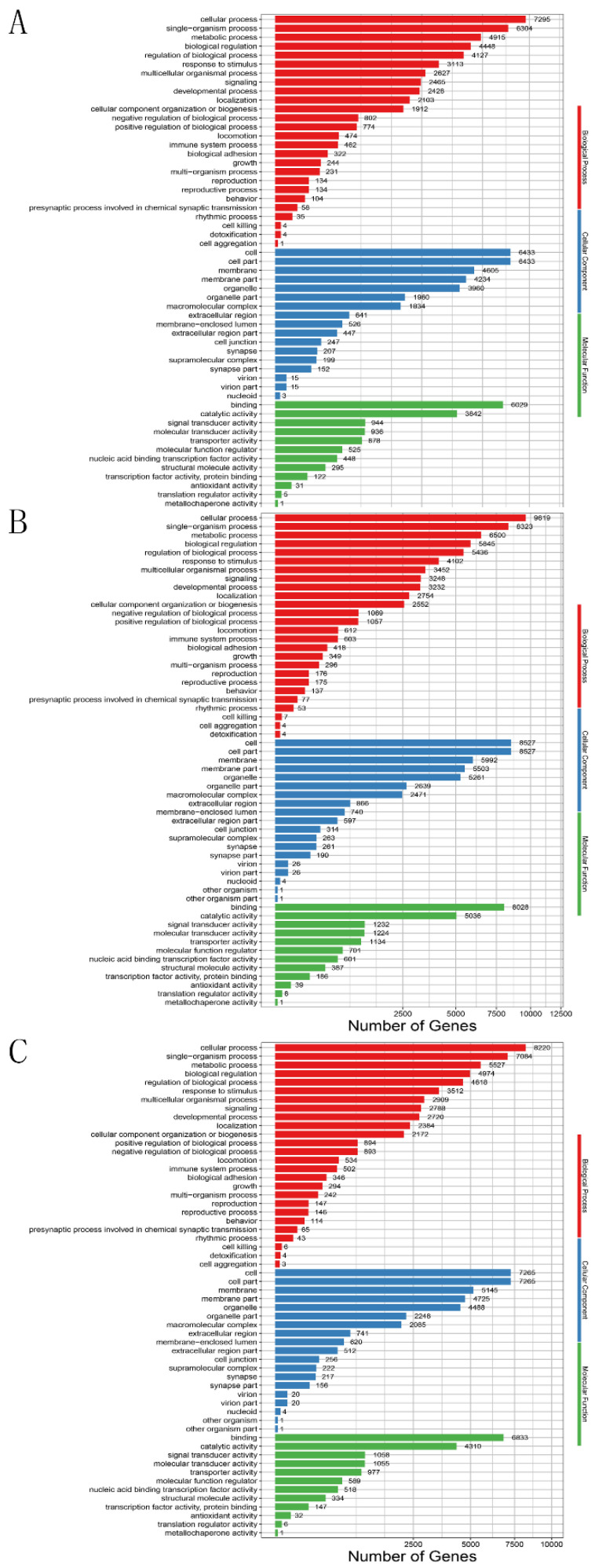
GO function classification of the differentially expressed genes comparison between the groups. (**A**) T0 vs. T1, (**B**) T0 vs. T2, (**C**) T0 vs. T3. The x-axis represents the number of genes and the y-axis represents different Gene Ontology (GO) term functional classification.

Next, GO and KEGG enrichment analyses were performed. GO enrichment analysis showed the similar results in the comparison of T0 vs. T1, T0 vs. T2 and T0 vs. T3. The target genes of miRNAs were identified for 55 enriched GO categories in terms of cellular component (CC), molecular functional (MF) and biological process (BP). Of these GO terms, integral component of membrane and membrane part in CC were significantly enriched (*p* ≤ 0.05). The biological process category of GO terms showed target genes were classified into 26 groups; the top of the most enriched GO term list were cellular process, biological regulation and single-organism process. The cellular component category of GO terms showed target genes were classified into 17 groups, the top of the most enriched GO term list were membrane, macromolecular complex and organelle. In the category of molecular function, binding, catalytic activity and signal transducer activity were the most significantly enriched with respect to 12 groups (Figure 3).

**Figure 4:**
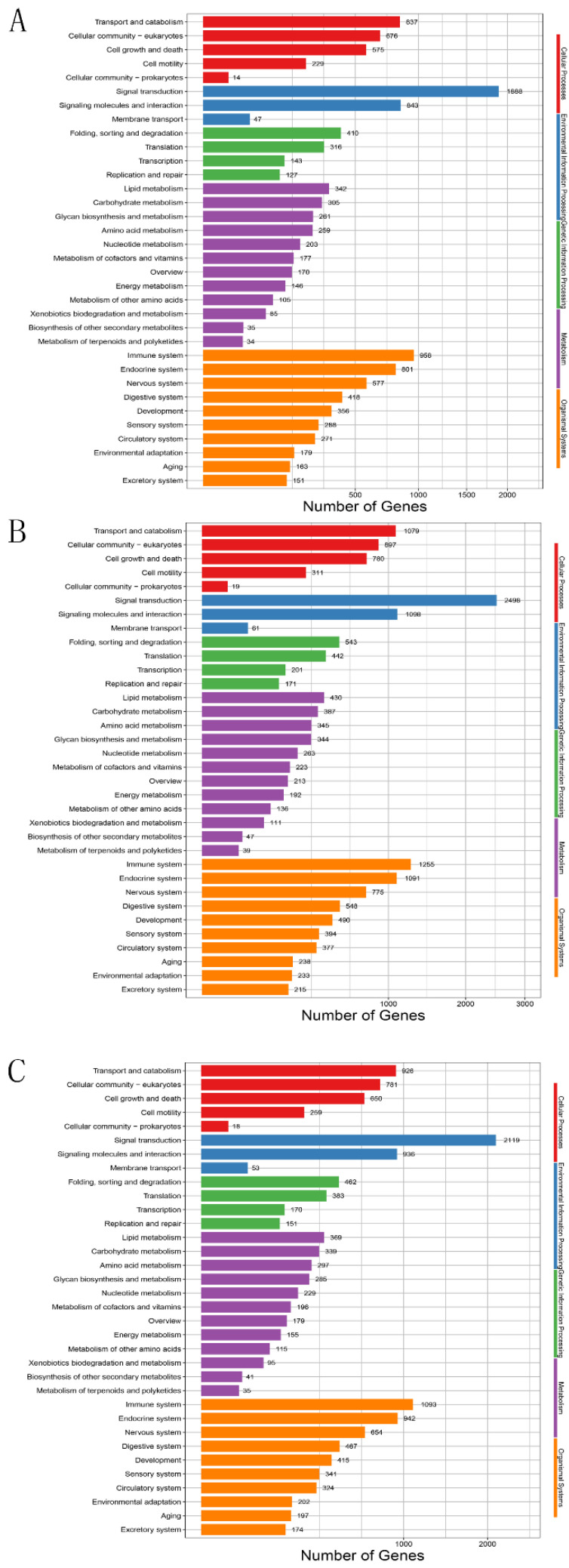
KEGG enrichment analysis of differentially expressed genes in different groups. (**A**) T0 vs. T1, (**B**) T0 vs. T2, (**C**) T0 vs. T3. The x-axis represents the number of genes and the y-axis represents different KEGG pathways.

According to the results of KEGG enrichment analysis, T0 vs. T1, T0 vs. T2 and T0 vs. T3 also showed the same enrichment trend. The 34 most significantly enriched pathways involving in 12,389, 16,446 and 14,052 target genes were divided into five clusters, respectively. The top five significantly enriched KEGG pathways include transport and catabolism, cell growth and death, immune system, signal transduction and lipid metabolism (Figure 4, Tables S2–S4).

## 4. Discussion

Hypoxia is a key factor limiting the survival rate of silver carp with high cultivation density. So far, there have been many studies on the response mechanism of silver carp under hypoxia stress [7]. Since miRNA was first discovered in *Caenorhabditis elegans,* increasing studies have confirmed that miRNA played an important role in the response of many aquatic organisms to stress [23]. However, the involvement of miRNAs in hypoxia tolerance in silver carp has not been extensively studied [2]. Therefore, we used the Illumina Noveseq high-throughput sequencing platform to study the small RNA in the mixed samples of heart, brain, liver and gill of silver carp under different levels of hypoxia stress, in order to lay a foundation for the further study of the regulatory role of miRNAs in the hypoxic stress response of silver carp. 

A total of 229 known miRNAs and 391 putative novel miRNAs were identified, which provided important information for further study of the mechanism of miRNAs in silver carp. By comparing the four hypoxic treatment groups, differentially expressed miRNAs upon hypoxia exposure were identified. Many of these miRNAs have been shown to be closely related to the regulation of hypoxic stress in aquatic organisms. Under hypoxia stress, 14 differentially expressed miRNAs were found in medaka (*Oryzias latipes*), and miR-204-5p was identified to be able to regulate apoptosis-related genes [24,25]. In this study, miR-204-5p showed an obviously high expression level, which suggested that miR-204-5p played an important role in regulating apoptosis of silver carp under hypoxia stress. The brain, liver and gonad of marine medaka were used as research materials, and high-throughput miRNA group sequencing was carried out [26]. It was found that the expression of let-7a, miR-122 and miR-9-3p was significantly down-regulated in female marine medaka under hypoxia, while miR-2184 was significantly up-regulated [27]. However, so far, there were few studies on the expression of miRNAs in silver carp under hypoxia stress. 

In the experiment of cerebral ischemia and hypoxia in rats, let-7f was found to be the upstream regulatory factor of NDRG3. When the expression of let-7f increased, the expression of NDRG3 decreased. Under hypoxic conditions, stable NDRG3 protein could promote angiogenesis and cell growth by activating the Raf-ERK pathway [28]. The experimental results confirmed the new mechanism of let-7f regulating the expression of NDRG3. The miR-125a has also been proven to play an important role in regulating animal-life activities under hypoxia stress [27]. In the study of the effect of hypoxia and reoxygenation on rat cardiomyocytes, miR-125a-5p can regulate the scavenger receptor B1 (Scarb1) gene, and the expression of miR-125a-5p in cardiomyocytes was affected by hypoxia and reoxygenation. When the expression of miR-125a-5p was inhibited, the expression of Scarb1 was significantly up-regulated, which played a protective role in cardiomyocytes [21]. MiR-146a has been confirmed to be involved in the occurrence and progression of a variety of cancers and played a key role in cell apoptosis [19]. The studies on miR-146a under hypoxia stress mainly focus on some human diseases. In the pathogenesis of osteoarthritis, it is confirmed that miR-146a played a key role in the autophagy of chondrocytes under hypoxia. The experimental results showed that miR-146a can promote autophagy by inhibiting the expression of Bcl-2 under hypoxia [29]. 

Except for the above differentially expressed miRNAs, other miRNAs with high expression levels such as dre-miR-143 and dre-miR-21 also played an important role in regulating the life activities of fish under hypoxia stress [30]. In the physiological adaptation of Atlantic cod (*Gadus morhua*) and Pacific whiteleg shrimp (*Litopenaeus vannamei*) under hypoxia stress, it was found that miR-143 could directly target the key enzyme-hexokinase in the mediation of glycolysis pathway, while hexokinase could accelerate glycolysis and produce ATP under hypoxia conditions [31]. In the study of the effect of hypoxia-reoxygenation on hepatocyte injury, miR-21 has been found to inhibit the activity of lactate dehydrogenase (LDH), thereby reducing the apoptosis of liver cells, and ultimately reducing the damage of liver cells [32,33,34]. In this study, the high expression of miR-21 was speculated to play an important regulatory role in hypoxia stress response of silver carp. 

GO and KEGG enrichment analysis of differentially expressed miRNAs target genes showed that differentially expressed miRNAs target genes were mainly involved in the regulation of hypoxia stress in silver carp through biological regulation, growth and death, immune system and signal transduction. In addition, relevant study has also found that miRNA played an important role in fat metabolism in extreme environments, which wasconsistent with the significant enrichment of lipid metabolism pathways in this study [35]. The results showed that under hypoxia conditions, most of the differentially expressed target genes of miRNAs were related to cell apoptosis, immunity and lipid metabolism [36,37]. This study explores the expression of miRNA in silver carp under different levels of hypoxia stress, and provides the reference for carrying out miRNA-target gene interaction in the later stage, improving the survival ability of silver carp under hypoxia environment.

## 5. Conclusions

Overall, the miRNAs expression of silver carp under different hypoxia stress levels was studied by high-throughput sequencing technology, which proved that miRNAs could be involved in the hypoxia stress of silver carp. Many miRNAs involved in the regulation of hypoxia in aquatic organisms, such as miR-204-5p, miR-143 and miR-125a-5p, have been identified. In addition, the target of miRNAs were predicted and functionally by GO and KEGG analysis, suggesting that these miRNAs may play a role in cell apoptosis and immunity. Our results provided new insights to further study the miRNAs regulatory mechanisms and molecular characteristics of hypoxia response in silver carp.

## Figures and Tables

**Table 1:** Primers used in qRT-PCR for miRNA expression validation.

miRNAs	Primer	Primer Sequence (5′-3′)
dre-let-7f	Loop	GTCGTATCCAGTGCAGGGTCCGAGGTATTCGCACTGGATACGACAACTAT
	F	GCGCGCTGAGGTAGTAGATTGT
	R	GCAGGGTCCGAGGTATTC
dre-miR-125a	Loop	GTCGTATCCAGTGCAGGGTCCGAGGTATTCGCACTGGATACGACCACAGG
	F	GCGCTCCCTGAGACCCTTAA
	R	GCAGGGTCCGAGGTATTC
dre-miR-216b	Loop	GTCGTATCCAGTGCAGGGTCCGAGGTATTCGCACTGGATACGACTCACAG
	F	GCGCGCTAATCTCTGCAGGCAA
	R	GCAGGGTCCGAGGTATTC
dre-miR-724	Loop	GTCGTATCCAGTGCAGGGTCCGAGGTATTCGCACTGGATACGACAACAGT
	F	GCGCGCTTAAAGGGAATTTGCG
	R	GCAGGGTCCGAGGTATTC
dre-miR-103	Loop	GTCGTATCCAGTGCAGGGTCCGAGGTATTCGCACTGGATACGACTCATAG
	F	GCGCAGCAGCATTGTACAGGG
	R	GCAGGGTCCGAGGTATTC
dre-miR-146a	Loop	GTCGTATCCAGTGCAGGGTCCGAGGTATTCGCACTGGATACGACCCATCT
	F	GCGCGCTGAGAACTGAATTCCAT
	R	GCAGGGTCCGAGGTATTC
dre-miR-27b-5p	Loop	GTCGTATCCAGTGCAGGGTCCGAGGTATTCGCACTGGATACGACTGTTCA
	F	GCGCGAGAGCTTAGCTGATTGG
	R	GCAGGGTCCGAGGTATTC
dre-miR-124-3p	Loop	GTCGTATCCAGTGCAGGGTCCGAGGTATTCGCACTGGATACGACTTGGCA
	F	GCGCTAAGGCACGCGGTGAA
	R	GCAGGGTCCGAGGTATTC
dre-miR-30e-5p	Loop	GTCGTATCCAGTGCAGGGTCCGAGGTATTCGCACTGGATACGACCTTCCA
	F	GCGCGCTGTAAACATCCTTGAC
	R	GCAGGGTCCGAGGTATTC
dre-miR-338	Loop	GTCGTATCCAGTGCAGGGTCCGAGGTATTCGCACTGGATACGACCAACAA
	F	GCGCGCTCCAGCATCAGTGATT
	R	GCAGGGTCCGAGGTATTC
dre-miR-30d	Loop	GTCGTATCCAGTGCAGGGTCCGAGGTATTCGCACTGGATACGACCTTCCA
	F	GCGCTGTAAACATCCCCGAC
	R	GCAGGGTCCGAGGTATTC
dre-miR-734	Loop	GTCGTATCCAGTGCAGGGTCCGAGGTATTCGCACTGGATACGACCGGTAC
	F	GCGCGCGTAAATGCTGCAGAATC
	R	GCAGGGTCCGAGGTATTC
dre-miR-17a-5p	Loop	GTCGTATCCAGTGCAGGGTCCGAGGTATTCGCACTGGATACGACTACCTG
	F	GCGCGCCAAAGTGCTTACAGTG
	R	GCAGGGTCCGAGGTATTC
dre-miR-16a	Loop	GTCGTATCCAGTGCAGGGTCCGAGGTATTCGCACTGGATACGACCACCAA
	F	GCGCGCTAGCAGCACGTAAATA
	R	GCAGGGTCCGAGGTATTC
dre-miR-16b	Loop	GTCGTATCCAGTGCAGGGTCCGAGGTATTCGCACTGGATACGACCTCCAA
	F	GCGCGCTAGCAGCACGTAAATA
	R	GCAGGGTCCGAGGTATTC
novel-517	Loop	GTCGTATCCAGTGCAGGGTCCGAGGTATTCGCACTGGATACGACTGCTCA
	F	GCGCGCACCTACACTGTCTAC
	R	GCAGGGTCCGAGGTATTC
U6	Loop	AAAACAGCAATATGGAGCGC
	F	TGCTCGCTACGGTGGCACA
	R	AAAACAGCAATATGGAGCGC

Note: F stands for forward primers; R stands for reverse primers; Loop stands for stem-loop primers.

**Table 2:** Quality data of small RNA library.

Items	T0	T1	T2	T3
Total reads	26,475,225	26,143,905	22,941,194	28,984,723
Clean reads	25,558,042	25,542,062	19,466,537	28,030,238
Q20	99.65%	99.65%	99.64%	99.62%
Q30	98.98%	98.97%	98.94%	98.88%
GC content	48.38%	47.87%	49.12%	48.28%

Note: Sample raw sequencing data yield and quality statistics. Q20 represents a 1% chance that the base will be incorrectly determined; Q30 represents a 1‰ chance that the base will be incorrectly determined.

**Table 3:** Highly expressed miRNAs in different groups.

miRNAs	miRNA Sequence (5′-3′)	T0	T1	T2	T3
dre-miR-100-5p	AACCCGUAGAUCCGAACUUGUG	2,167,459	1,924,641	1,089,212	1,969,635
dre-miR-143	UGAGAUGAAGCACUGUAGCUC	1,113,425	641,659	2,373,465	1,570,961
dre-miR-101a	UACAGUACUGUGAUAACUGAAG	688,497	1,472,818	484,507	396,814
dre-miR-146a	UGAGAACUGAAUUCCAUAGAUGG	167,716	70,684	248,036	193,016
dre-miR-21	UAGCUUAUCAGACUGGUGUUGGC	416,516	318,134	327,475	347,802
dre-miR-22a-3p	AAGCUGCCAGCUGAAGAACUGU	757,583	492,558	1,363,640	664,397
dre-miR-26a-5p	UUCAAGUAAUCCAGGAUAGGCU	670,590	490,815	706,568	666,125
dre-miR-30d	UGUAAACAUCCCCGACUGGAAG	190,391	226,262	352,041	202,079
dre-miR-99	AACCCGUAGAUCCGAUCUUGUG	613,288	668,863	1,041,263	667,971
dre-let-7e	UGAGGUAGUAGAUUGAAUAGUU	216,109	443,662	160,268	439,320
dre-miR-30e-5p	UGUAAACAUCCUUGACUGGAAG	92,054	103,939	119,573	48,008
dre-miR-451	AAACCGUUACCAUUACUGAGUU	31,188	16,482	36,582	23,333
dre-miR-27b-3p	UUCACAGUGGCUAAGUUCUGCA	88,506	40,150	223,389	84,618

Note: Several miRNAs that highly expressed in four groups.

**Table 4:** Number of reads matched to various types of sequences.

Types	T0	T0 (Percent)	T1	T1 (Percent)	T2	T2 (Percent)	T3	T3 (Percent)
total	22,823,393	100.00%	24,310,475	100.00%	17,914,838	100.00%	25,405,520	100.00%
known_miRNA	16,902,843	74.06%	19,366,521	79.66%	13,751,148	76.76%	18,336,438	72.18%
rRNA	408,035	1.79%	201,337	0.83%	545,212	3.04%	981,912	3.86%
tRNA	2	0.00%	0	0.00%	8	0.00%	2	0.00%
snRNA	27,663	0.12%	19,041	0.08%	11,027	0.06%	17,458	0.07%
snoRNA	11,148	0.05%	8611	0.04%	36,446	0.20%	22,629	0.09%
repeat	1,506,607	6.60%	532,386	2.19%	154,534	0.86%	971,529	3.82%
novel_miRNA	132,493	0.58%	90,735	0.37%	94,536	0.53%	121,857	0.48%
exon: +	141,310	0.62%	60,768	0.25%	336,910	1.88%	170,306	0.67%
exon: −	112,890	0.49%	44,975	0.19%	272,386	1.52%	138,940	0.55%
intron: +	164,073	0.72%	103,326	0.43%	85,369	0.48%	157,480	0.62%
intron: −	89,759	0.39%	70,626	0.29%	50,486	0.28%	112,331	0.44%
other	3,326,570	14.58%	3,812,149	15.68%	2,576,776	14.38%	4,374,638	17.22%

Note: Distribution of different types of small RNA in each group.

**Table 5:** The number of up-regulated and down-regulated differentially expressed miRNAs.

	UP	T0	T1	T2	T3
DOWN					
T0			75	210	104
T1		55		183	100
T2		112	152		144
T3		60	95	186	

Note: The number of differentially expressed miRNAs was determined by pairwise comparison. Above the diagonal is the number of up-regulated differentially expressed miRNAs. Below the diagonal is the number of down-regulated differential miRNAs.

## Data Availability

The data presented in this study are available on request from the corresponding author.

## Supplementary Materials

The following are available online at www.mdpi.com/article/10.3390/ani11102917/s1, Figure S1: Volcano plot of differentially expressed miRNAs in T0 vs. T1, T0 vs. T2, T0 vs. T3, T1 vs. T2 and T2 vs. T3. The red and green dots represent up-regulated and down-regulated differentially expressed miRNAs, Figure S2: Box diagram of miRNA TPM distribution in different groups, Figure S3: Differential miRNA clustering heat map, Table S1: Summary of Novel miRNA identification results, Table S2: KEGG enrichment pathway of DEMs target genes (T1 vs. T2), Table S3: KEGG enrichment pathway of DEMs target genes (T1 vs. T3), Table S4: KEGG enrichment pathway of DEMs target genes (T2 vs. T3).
