# Supplementary material for: Identification of microRNAs in Silver Carp (Hypophthalmichthys molitrix) Response to Hypoxia Stress
Source: Animals (Basel). 2021 Oct 9;11(10):2917. doi: 10.3390/ani11102917 (PMC8696637; doi:10.3390/ani11102917)
Supplement: Supplementary file 1 [file animals-11-02917-s001.zip › Figure S1.pptx]

## Slide 1
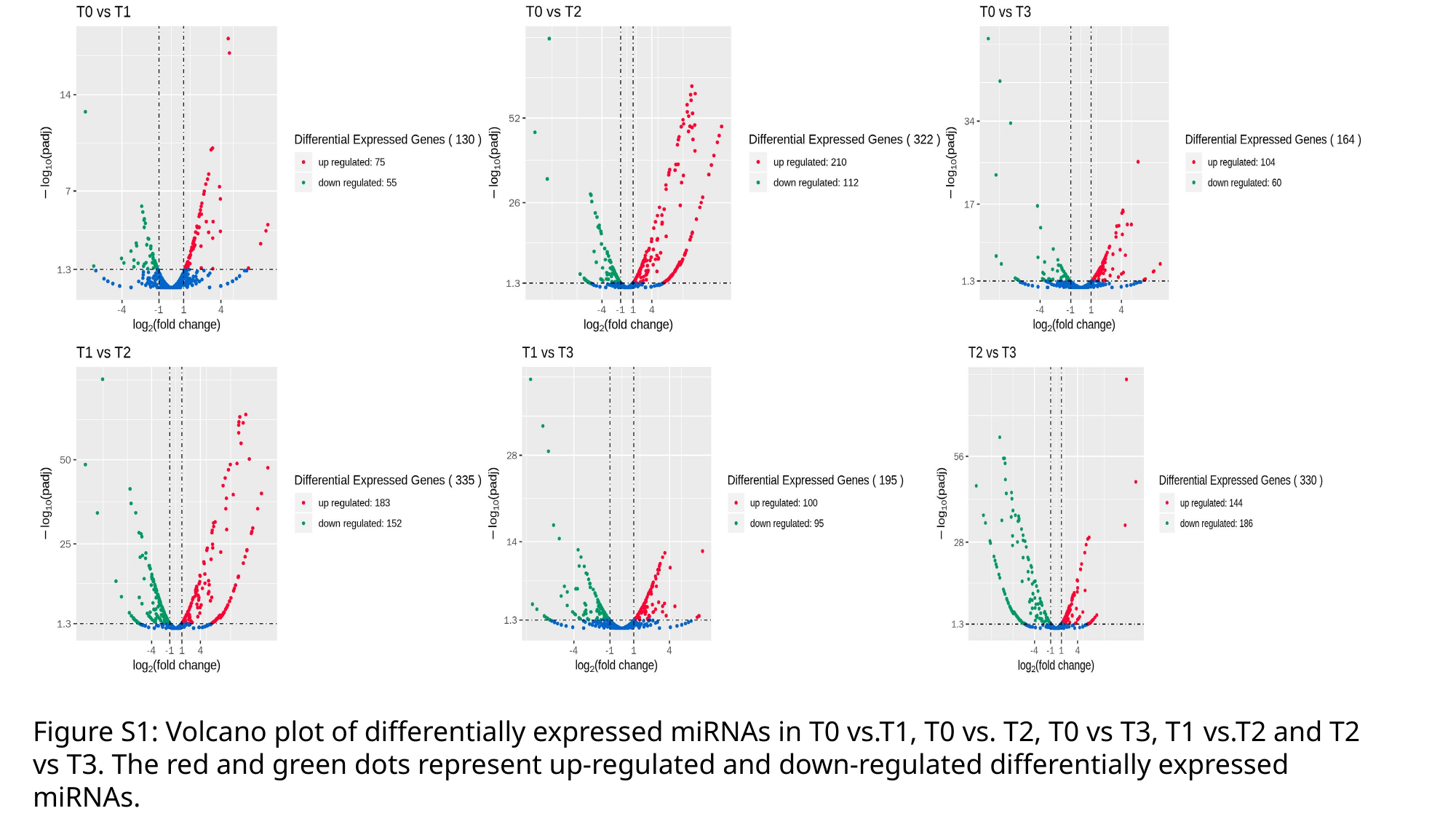

Figure S1: Volcano plot of differentially expressed miRNAs in T0 vs.T1, T0 vs. T2, T0 vs T3, T1 vs.T2 and T2 vs T3. The red and green dots represent up-regulated and down-regulated differentially expressed miRNAs.
