# Supplementary figures and images for: Identification of microRNAs in Silver Carp (Hypophthalmichthys molitrix) Response to Hypoxia Stress
Source: Animals (Basel). 2021 Oct 9;11(10):2917. doi: 10.3390/ani11102917 (PMC8696637; doi:10.3390/ani11102917)

## Slide 1
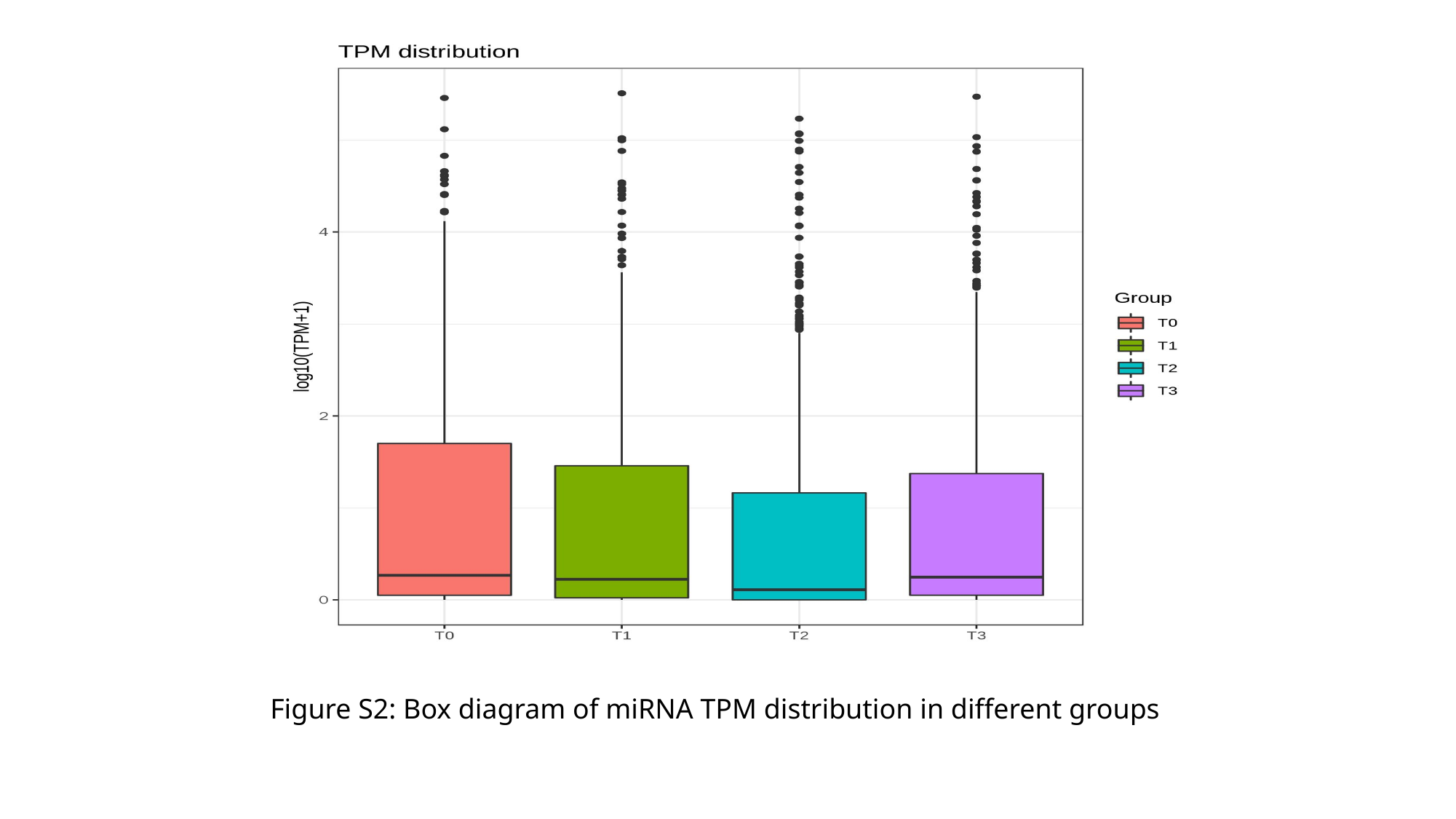

Figure S2: Box diagram of miRNA TPM distribution in different groups

Supplement: Supplementary file 1 [file animals-11-02917-s001.zip › Figure S2.pptx]

## Slide 1
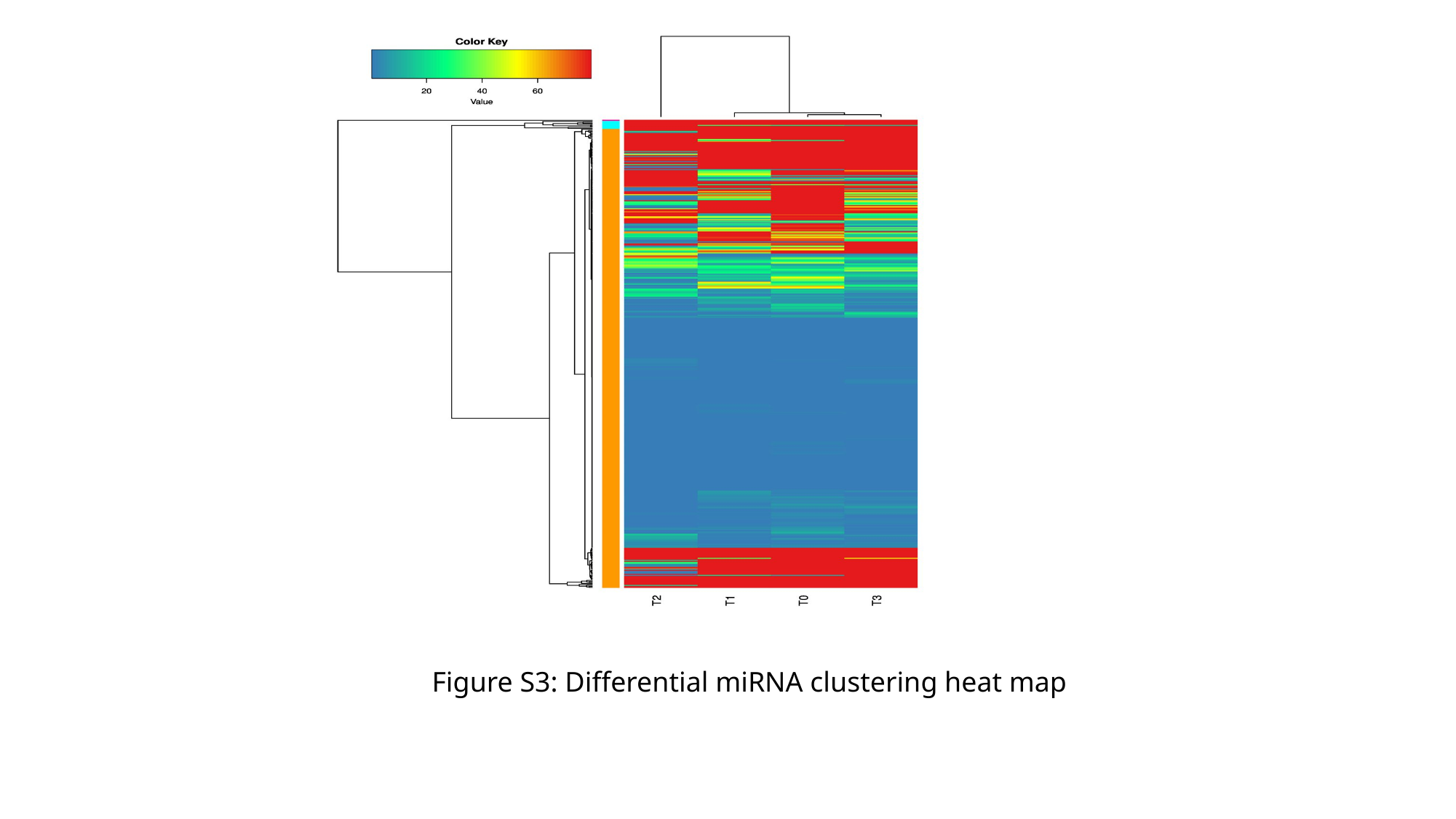

Figure S3: Differential miRNA clustering heat map

Supplement: Supplementary file 1 [file animals-11-02917-s001.zip › Figure S3.pptx]
